# Revisiting Unplanned Endotracheal Extubation and Disease Severity in Intensive Care Units

**DOI:** 10.1371/journal.pone.0139864

**Published:** 2015-10-20

**Authors:** Ming-Lung Chuang, Chai-Yuan Lee, Yi-Fang Chen, Shih-Feng Huang, I-Feng Lin

**Affiliations:** 1 Division of Pulmonary Medicine, Chung Shan Medical University Hospital, Taichung, Taiwan; 2 Department of Critical Care Medicine, Chung Shan Medical University Hospital, Taichung, Taiwan; 3 School of Medicine, Chung Shan Medical University, Taichung, Taiwan; 4 Department of Nursing, Chung Shan Medical University, Taichung, Taiwan; 5 Division of Respiratory Care, Chung Shan Medical University Hospital, Taichung, Taiwan; 6 Institute and Department of Public Health, National Yang Ming University, Taipei, Taiwan; D'or Institute of Research and Education, BRAZIL

## Abstract

Most reports regarding unplanned extubation (UE) are case-control studies with matching age and disease severity. To avoid diminishing differences in matched factors, this study with only matching duration of mechanical ventilation aimed to re-examine the risk factors and the factors governing outcomes of UE in intensive care units (ICUs). This case-control study was conducted on 1,775 subjects intubated for mechanical ventilation. Thirty-seven (2.1%) subjects with UE were identified, and 156 non-UE subjects were randomly selected as the control group. Demographic data, acute Physiological and Chronic Health Evaluation II (APACHE II) scores, and outcomes of UE were compared between the two groups. Logistic regression analysis was used to identify the risk factors of UE. Milder disease, younger age, and higher Glasgow Coma Scale (GCS) scores with more frequently being physically restrained (all p<0.05) were related to UE. Logistic regression revealed that APACHE II score (odds ratio (OR) 0.91, p<0.01), respiratory infection (OR 0.24, p<0.01), physical restraint (OR 5.36, p<0.001), and certain specific diseases (OR 3.79–5.62, p<0.05) were related to UE. The UE patients had a lower ICU mortality rate (p<0.01) and a trend of lower in-hospital mortality rate (p = 0.08). Cox regression analysis revealed that in-hospital mortality was associated with APACHE II score, age, shock, and oxygen used, all of which were co-linear, but not UE. The results showed that milder disease with higher GCS scores thereby requiring a higher use of physical restraints were related to UE. Disease severity but not UE was associated with in-hospital mortality.

## Introduction

Unplanned endotracheal extubation (UE) is an indicator of the quality of care in intensive care units (ICUs) [1,2] and is reported to cause subsequent complications[1,3,4,5,6,7,8,9,10,11]. The risk factors for UE include prolonged use of mechanical ventilatory support (MVS) [2,7,8,12], oral route of intubation [4,5], clearer consciousness [13,14], frequent use of physical restraint [3,13,15,16], and others [3,4,5,6,13]. However, some controversy exists with regards to a higher incidence of UE during sedation leading to paradoxical excitation[17], the non-use of physical restraints[18], the oral route for intubation not being a risk factor[12], and increases in in-ICU and in-hospital mortality[1,7].

Most reports in the literature regarding UE are matched case-control studies [3,5,7,8,15,17], and this design may introduce bias or diminish differences in matched factors [6,19] such as age and disease severity [3,7,8,17]. It therefore seems necessary to re-examine the controversial issues regarding UE. We hypothesized that age and disease severity per se governs the outcomes of UE. Therefore, the aim of this study was to identify the risk factors and the factors dictating outcomes of UE by only matching the duration of mechanical ventilation [6] between a case group and a control group, as the importance of matching the duration of mechanical ventilation between groups has been addressed by de Groot et al [6] (S1 File The number of subjects using ventilator before UE and the number of subjects of the corresponding matched group).

## Materials and Methods

### Study design

This was a case-control study with one UE subject to four randomly selected subjects without UE (non-UE). We did not enroll all non-UE patients as this was a huge group which would have caused an uneven allocation of subjects. Furthermore, sampling UE and non-UE subjects at a ratio from 1:2 to 1:4 has been supported by previous reports [6,7,8,17], with a 1:4 ratio being ideal because the power to differentiate between two groups reaches a plateau at this level [20]. We enrolled additional 5% of patient number in the control group to compensate possible missing data.

### Study setting

This study was conducted in three closed-system adult mixed ICUs (61-bed capacity) of a medical center in Taiwan (CSH-2014-A-032). The ICUs were staffed by qualified ICU physicians and senior residents who provided 24-hour in-unit care, and experienced nurses, nursing specialists, respiratory therapists, pharmacists, and dietitians. The patient-ICU physician ratio was approximately 10:1, and the patient-nurse ratio was 2–2.5:1 per 24 hours.

### Subjects

All adult patients were eligible if they required an artificial airway for MVS from January 1 to December 31, 2012. The patients with a tracheostomy were excluded from the study. The patients with UE were defined as the case group, and these patients were routinely reported as per the standard practice of the Patient Safety Management Policy of the hospital, and routinely recorded by nurses and respiratory therapists. In general, UE incidents are much less frequent in an ICU. Therefore, to avoid unevenly allocating the two groups, four patients without UE were selected using a random number generator for one UE subject. No consent was required as the study design that the data were analyzed anonymously was approved by the Institutional Review Board of Chung Shan Medical University (No. CS 13072). The experimental research was conducted in compliance with the Declaration of Helsinki. Within the 1-year study period, 3427 subjects were admitted to the ICUs, including 1775 (52%) subjects who required oral endotracheal intubation for MVS.

### Definitions

Unplanned extubation was defined as the endotracheal tube being removed in an unplanned manner by the patient. This included deliberate endotracheal self-extubation where the endotracheal tube was deliberately removed by the patient. The UE incidence rate was defined as the ratio of the number of unplanned extubations and the number of times MVS was instituted in a given time period [13]. The UE incidence density was defined as the ratio of the number of unplanned extubations and the number of times MVS was instituted every 100 days [13].

### Data collection

Data were retrieved from the database of the hospital’s computer system with the permission of the Institutional Review Board of the hospital. The data included demographics, admission diagnosis (disease entities), Acute Physiological and Chronic Health Evaluation II (APACHE II) score, Glasgow Coma Score (GCS), ICU management, use of sedation, use of physical restraints, length of MVS use, and length of ICU and hospital stay. MVS settings and cardiopulmonary responses were recorded within six hours before extubation and were collected from medical records. Data on arterial blood gas (ABG) were also collected from medical records. Because the UE events could not be predicted, we did not always have simultaneous ABG data. If the ABG data were obtained 3 days or more before the event, the data were deemed to be missing. The ABG data, cardiopulmonary responses and MVS settings of the non-UE group were collected at the corresponding time of the UE (S1 File The number of subjects using ventilator before UE). To ensure that the number of UE events was correct, the events were double-checked from the incident reporting system of the computer system, the nursing records, and respiratory therapist records.

### Statistical analysis

Data were presented as mean ± standard deviation (SD) or median (interquartile) for continuous variables, or as frequency (percentage) for categorical variables. Unpaired t-tests or Mann-Whitney tests were used to compare the means or medians of variables between two independent groups. The chi-square test was used for categorical variables, and Fisher’s exact test was used when the expected number in any cell was <5. Logistic regression was conducted to identify the clinical characteristics associated with UE. Cox regression was conducted to identify the risk factors for mortality. All tests were two-sided and statistical significance was set at a *p* value less than 0.05. A *p* value of less than 0.1 but more than 0.05 was considered to indicate a trend in difference [21]. All statistical analyses were performed using SAS version 9.3 (SAS Institute Inc., Cary, NC).

## Results

**Fig 1:**
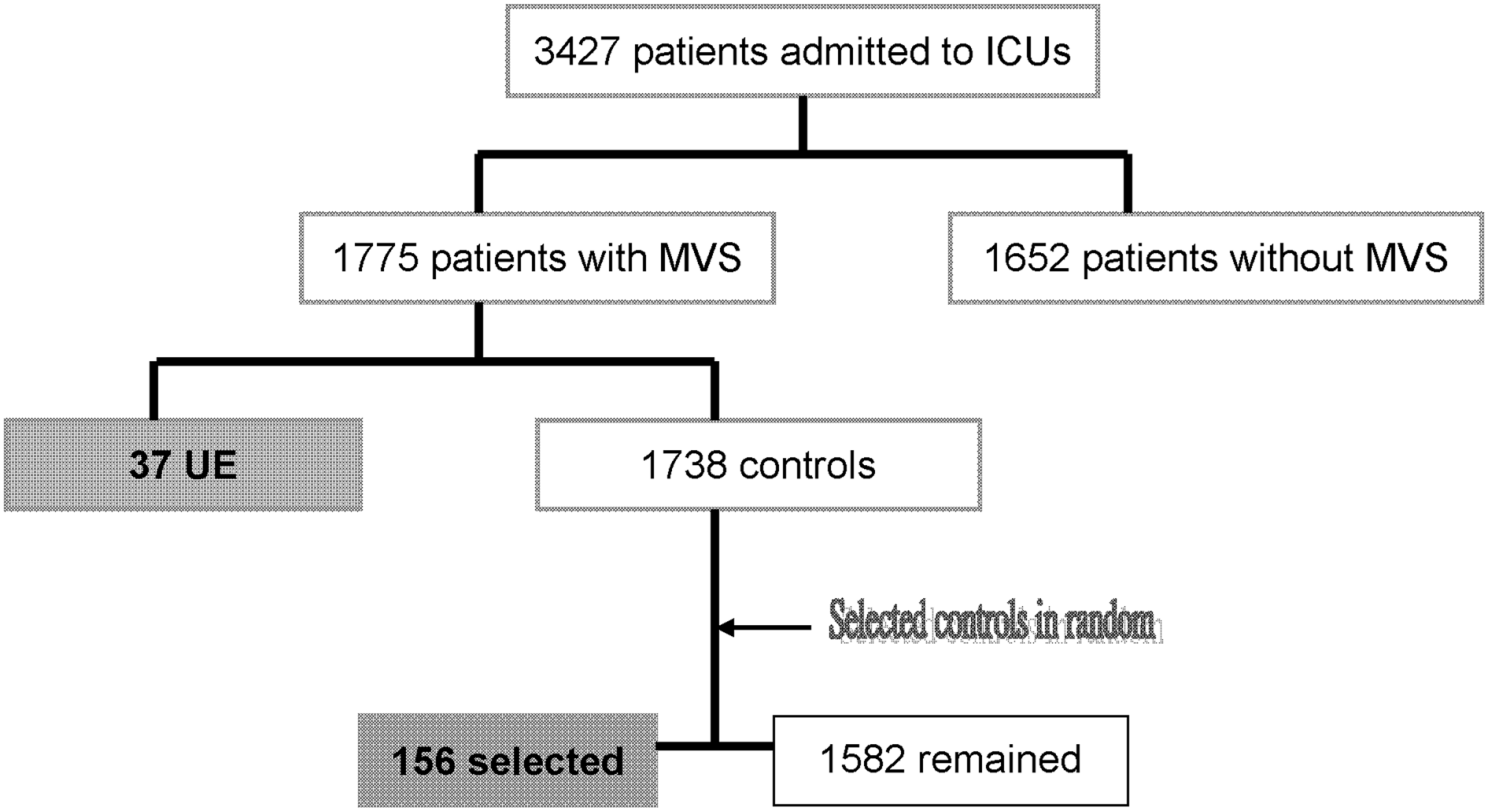
Flow Diagram. The frequencies of annual admission to intensive care units (ICUs), mechanical ventilatory support (MVS), and unplanned extubation (UE). Controls are the subjects without UE.

Thirty-seven UEs occurred (incidence rate 2.1%, Fig 1), with no difference among the ICUs (χ^2^ test, *p* = 0.1). The data from the three ICUs were then pooled for analysis. The incidence density of UE in the ICUs was 4.3‰, and no recurrence of UE was noted.

### Clinical characteristics of the patients with unplanned extubation

The UE patients were significantly younger and had lower APACHE II scores and higher GCS scores at admission (Table 1, all *p*<0.05) than the non-UE patients. The UE patients had fewer catheterizations (*p*<0.01), but were more frequently physically restrained (*p*<0.001). They also had lower incidences of infections, acute respiratory failure, renal failure, shock, and hypoxic encephalopathy (all *p*<0.05) (Table 2). Multiple logistic regression analysis revealed that APACHE II scores (odds ratio (OR) 0.91, *p*<0.01), respiratory infections (OR 0.24, *p*<0.01), more frequent use of physical restraints (OR 5.36, *p*<0.001) and certain specific diseases (OR 3.79–5.62, *p*<0.05-<0.01) were associated with the incidence of UE (Table 3).

**Table 1:** Baseline characteristics and selected outcomes.

	UE, n = 37	non-UE, n = 156	*P* value
Age, year	65±19	71±15	0.04
Body mass index, kg/m^2^	24.3±4.6	22.9±4.7	0.09
Sex, M:F	24:13	85:63*	NS
APACHE II	19.6±5.8	23.2±8.4	<0.001
Glasgow Coma Scale^†^	10±3.2	8.6±3.5	<0.01
Foley catheter, yes/no	27/10	120/14*	0.01
CVC, yes/no	19/18	108/28*	<0.001
Sedation^#^, yes/no	26/11	111/42*	NS
Restraint, yes/no	27/10	63/90*	<0.001
VEST^TM^, yes/no	11/26	35/118*	NS

Abbreviations: UE: unplanned extubation; APACHE II, Acute Physiological and Chronic Health Evaluation II; CVC, central venous catheter; Glasgow Coma Scale: score measured 1–7 days before and nearest the unplanned extubation; VEST^TM^, a high frequency chest wall oscillator; NS, not significant.

^#^Sedation use during this admission;

*Some missing data.

^†^After excluding 17 patients with hypoxic encephalopathy in the non-UE group, the difference in Glasgow coma scores between the two groups remained significant (10±3.2 versus 7.5±3.3, p < .0001)

**Table 2:** Univariate disease entities.

Disease Category	Classification	UE, n = 37		non-UE, n = 156		χ^2^
		Yes	No	Yes	No	p value
Infection	Respiratory	16	21	128	28	<0.0001
	Septicemia	13	24	86	70	0.03
	TB or chronic infection	0	37	8	148	0.16
Cardiovascular	Arrhythmia	12	25	25	131	0.02
Respiration	Acute respiratory failure	23	14	132	24	<0.01
Kidneys	Renal failure	9	28	80	76	0.001
Shock		8	29	63	93	0.03
Neurological	Hypoxic encephalopathy^†^	0	37	17	139	0.04
Poisoning		2	35	0	156	<0.01
Trauma		6	31	12	144	0.11

Abbreviations: UE: unplanned extubation; TB, tuberculosis;

^†^After excluding 17 patients with hypoxic encephalopathy in the non-UE group, the difference in Glasgow coma scores between the two groups remained significant (10±3.2 versus 7.5±3.3, p < .0001)

**Table 3:** Multiple logistic regression analysis for unplanned extubation.

Variables	Odds Ratio	95% CI	P value
APACHE II	0.91	(0.85, 0.97)	<0.01
Physical restraint	5.36	(1.99, 14.46)	<0.001
Pleural disorders	5.62	(1.61, 19.64	<0.01
Coronary artery disease	5.31	(1.30, 21.67)	0.02
Urinary tract infection	3.79	(1.07, 13.39)	0.04
Respiratory infection	0.24	(0.09, 0.69)	<0.01

APACHE II: Acute Physiological And Chronic Health Evaluation II; CI: confidence interval

The MVS settings in the UE group tended to more frequently include pressure-support mode (*p* = 0.055) with a significantly lower F_I_O_2_ (*p*<0.01) and respiratory rate (*p* = 0.01), while the non-UE group tended to be set to the assist/control mode (Table 4). The UE group had lower peak airway pressure (*p* = 0.04) and pulse rate (*p* = 0.05), and tended to have lower airway resistance (*p* = 0.07).

**Table 4:** Mechanical ventilatory support (MVS) settings and cardiopulmonary responses before unplanned extubation (UE) (mean±SD).

Variables	UE, n = 37	non-UE*, n = 156	*p* value
Settings			
MVS, no. AC/no. PSV mode	21/16	93/34	0.055
F_I_O_2_, %	40±14	50±25	<0.01
Pressure set, cm H_2_O	16±7	18±7	NS
PEEP, cm H_2_O	5±2	6±2	0.08
Respiratory rate, b/min	12±2	14±4	0.01
Patients’ responses			
P_peak_, cm H_2_O	21±6	24±6	0.04
Respiratory rate, b/min	17±4	18±6	NS
I:E	1: 3.2±1	1: 2.9±1.4	NS
Tidal volume, L	.53±.14	.52±.11	NS
Minute ventilation, L/min	9±3.7	9.4±3	NS
Lung compliance, ml/cm H_2_O	45±13	42±18	NS
Resistance, cm H_2_O/L/min	11±5	15±6	0.07
Systolic BP, mm Hg	132±24	124±26	NS
Diastolic BP, mm Hg	66±17	61±15	NS
Pulse rate, b/min	85±15	94±24	0.05

Abbreviations: no. AC, patient number used assisted control; no. PSV, patient number used pressure support ventilation; F_I_O_2_, fraction of inspired oxygen; PEEP, positive end-expiratory pressure; P_peak_, peak airway pressure; I:E, inspiratory time and expiratory time ratio; BP, blood pressure; NS, not significant.

*Data measured on the corresponding day as the UE group with some missing data.

### Outcomes of unplanned extubation and mortality analysis

The UE group had a longer ICU stay (*p* = 0.04), however, there were no significant differences in the length of MVS use and hospital stay (both *p*>0.05) (Table 5). In the UE group, the in-ICU mortality rate was significantly lower (*p*<0.01), and the in-hospital mortality rate tended to be lower (*p* = 0.08). The risk factors for mortality included older age, higher APACHE II score, shock, and using a higher F_I_O_2_, but not UE (S2 File Hazard ratio). Age, shock, and F_I_O_2_ use were co-linear to APACHE II scores.

**Table 5:** Outcomes of unplanned extubation (UE).

Variables	UE, n = 37	non-UE*, n = 156	*p* value
ICU LOS, days	11.4±8.3	8.3±6.8	0.04
MVS before UE, days	5.2±4	-	-
MVS, total days	9.3±8.5	7±6.3	NS
Hospital LOS, days	24.3±26.9	16.5±24.6	NS
In-ICU mortality, %	5.4	27.4	<0.01
In-hospital mortality, %	24.3	39.7	0.08

Abbreviations: ICU, intensive care unit; LOS, length of stay; MVS, mechanical ventilatory support; UE, unplanned extubation; NS, not significant

*Some missing data.

## Discussion

In the present study, we found that the subjects with UE were younger, had milder disease severity and higher GCS scores (thereby being more frequently physically restrained). They also had lower in-ICU mortality and a lower trend in in-hospital mortality. The risk factors for in-hospital mortality were age, APACHE II score, shock, and using a higher F_I_O_2_, but not including UE. These risk factors were all related to APACHE II score (*p* = 0.014–0.0018) (S2 File Hazard ratio).

### Risk factors for unplanned extubation

When identifying the risk factors for UE, it might be inappropriate to control for age, severity of illness, and diagnostic category as this would reduce the significance of their contribution. Many risk factors for UE have been identified including higher GCS score [3,4,5,13,14] and more frequent use of physical restraints [3,13,15,16]. As most previous studies have controlled for disease severity, disease severity has rarely been considered a risk factor for UE [3,7,8,17]. In this study, we did not control for age or disease severity and they appeared to be risk factors for UE, consistent with a previous report [22]. Male predominance was not a risk factor in this study as male predominance existed in both groups, which is consistent with previous reports [6,14]. Of note, oral intubation was the routine method for endotracheal intubation in our institution, and the incidence rate of UE was only 2.1%, which is low as compared to that reported in the literature varying between 0.5% and 35.8% in the recent years [13].

Using physical restraints with sedation for agitated patients or those with relatively clearer consciousness has been recommended [15]. However, most studies report using a sedation regimen without knowing the consciousness level [4,15,17,23]. In this study, the subjects with UE had higher GCS scores at admission and before the UE events (UE versus non-UE 11.7±2.6 versus 9.8±10.8, p = .04) and received inadequate sedation for agitation so that they were physically restrained more frequently (Table 1). This does not mean the being physically restrained per se is a risk factor of UE, and our findings are consistent with a previous report [14].

Respiratory disorders have been reported to be a risk factor for UE [4,13]. In contrast, this study showed that respiratory infections occurred less frequently (OR 0.24, *p*<0.01), although pleural effusion (OR 5.62, *p*<0.01), coronary artery disease (OR 5.31, *p*<0.05), and urinary tract infections (OR 3.79, *p*<0.05) occurred more frequently in the UE group (Table 3). This may be attributed to disease severity, as the subjects with respiratory infections and respiratory failure had higher APACHE II scores than those without (*p* = 0.01), and the subjects with pleural effusion had lower APACHE II scores than those without (*p* = 0.1) (S3 File Acute physiological and chronic health evaluation II scores).

The ventilator settings and the patient’s cardiopulmonary response to mechanical ventilation may also represent disease severity. In this study, the use of positive end-expiratory pressure levels tended to be lower, F_I_O_2_ and respiratory rate were significantly lower (both *p*≤0.01), and cardiopulmonary function seemed to be better in the UE group than in the non-UE group (both *p*≤0.05) (Table 4). These findings are consistent with a previous report [14].

### Outcomes of unplanned extubations and mortality analysis

It has been reported that complications of UE include prolonged use of MVS [8], thereby extending the ICU and hospital stay [3,8]. However, patients with UE have also been reported to have a marginally [6] or significantly [5] shorter duration of MVS use and ICU stay, and no difference in [6] or shorter [5] length of hospital stay. The present study showed that the UE group had a longer ICU stay (*p* = 0.04), however the total duration of MVS use and hospital stay were not different between the two groups (both *p* = NS) (Table 5). This is probably due to prolonged observation for the patients with successful UE in the ICU (usually 1–2 days more) in the current study, the on-going use of MVS for the patients with unsuccessful UE [1,5,7,8], and the short stay in the ICU for the patients without UE if they died earlier. The cause and effect between UE and the prolonged ICU stay is controversial. However, it is noted that the ICU stay of the unsuccessful UE versus the successful UE is 17±9.2 days versus 7.5±4.7 days (p < .01). The duration of MVS use of the unsuccessful UE versus the successful UE is 15.7±9.8 days versus 5±3.4 days (p < .001). The discrepancies between all of the previous reports including the current study may be attributed to the heterogeneity of the subjects, the disease entities and severity, and the different proportion of patients with unsuccessful UE.

The in-ICU mortality rate was extremely low in the UE group of this study, in contrast to previous reports [1,4,5,7,8,9,10,11]. However, the in-hospital mortality rate had only a trend in difference between the UE group and non-UE group (*p* = 0.08, Table 5). This discrepancy may be due to different health policies for patient deposition in different countries. The length of ICU stay in Taiwan is suggested to be ≤21 days and encouraged to be ≤14 days. When the length of stay in an ICU approaches 14 days, clinicians usually transfer the subjects to step-down units (i.e., respiratory care center) for weaning from mechanical ventilation unless there are contraindications. Some risk factors of in-hospital mortality were identified in the current study, however all were co-linear to APACHE II score.

### Study limitations

This study did not enroll all non-UE patients in design as a control group for comparison as the number of this group of patients was huge. Case-control studies are appropriate for a low incidence rate of events regardless of the retrospective [3,5,7,8,14,17] or prospective design [6] and may save tremendous labor. However, matching selected variables may diminish differences in the variables between groups [6,19], as can be seen in previous matched disease severity case-control studies reporting that disease severity was not a risk factor for UE [3,7,8,17]. Observational studies on a single group of UE subjects without a control group are less appropriate, as this may introduce bias [12,24,25,26].

The dose of sedative agents administered could not be accurately presented, as the nursing staff frequently adjusted the dose based on the sedation status of the patients according to Richmond Agitation-Sedation Scale (RASS) scores of -2 during the daytime and -3 at nighttime and the poorly complied with the RASS target [27]. There were no differences in midazolam maleatel or propofol-lipuro use on the day of UE or virtual day of UE between UE group and non-UE group, retrospectively (5.2 ±2.4 vs. 5.3±3.1 mg/h or 64±23 vs. 69±40 mg/h, respectively, both p = NS). However, due to the retrospective design of the study, we cannot exclude the mechanism of UE like that patients with milder disease severity or the patients with lower setting of MVS were less sedated thereby having higher GCS score and agitation and requiring more physical restraints. In addition, we did not measure the RASS and APACHE II scores simultaneously with the incident. It could be argued that the 17 patients with hypoxic encephalopathy in the non-UE group confounded the GCS scores to be a risk factor for UE (Table 2). However, after exclusion of these 17 patients, the difference in GCS scores between the UE group and non-UE group remained significant (10±3.2 versus 7.5±3.3, *p*<0.0001). Despite following the weaning protocol of our institution (S4 File The weaning protocol), the UE incidents still could not be avoided, suggesting that team work [28] to early identify those at high risk for UE to reduce incidents is necessary. There were no cases of recurrent UE in this study, so our findings may not be applicable to institutions where recurrent UE occurs frequently [1]. Finally, UE-related nosocomial infections have been reported [1,15], however these data are not presented in this study as this was not the primary aim. Indeed, UE-related nosocomial infections may prolong the ICU stay [1].

## Conclusions

The risk factors for UE were lower age, milder disease and higher GCS score (thereby having a more frequent use of physical restraints). To early identify those at high risk for UE to reduce the incidents is responsible for each care-giver. UE may prolong ICU stay but is not associated with in-hospital mortality. APACHE II score is the most important factor associated with in-hospital mortality.

## Supporting Information

S1 Dataset Minimal data set. The original data set of the study in part.
(XLS)

S1 File The number of subjects using ventilator. The number of subjects in each group by the days of mechanical ventilation use before the unplanned extubation (UE) occurred.
(DOC)

S2 File A. Hazard Ratio for mortality using Cox regression. B. Acute physiological and chronic health evaluation II scores (APACHE II) and the related factors. (DOCX)

S3 File Acute physiological and chronic health evaluation II scores of various diseases. The scores (mean±SD) of various disease entities.
(DOC)

S4 File The weaning protocol. The process of weaning from the MVS used in this institution.
(DOC)
