# Supplementary material for: Revisiting Unplanned Endotracheal Extubation and Disease Severity in Intensive Care Units
Source: PLoS One. 2015 Oct 20;10(10):e0139864. doi: 10.1371/journal.pone.0139864 (PMC4617893; doi:10.1371/journal.pone.0139864)
Supplement: S1 File — The number of subjects in each group by the days of mechanical ventilation use before the unplanned extubation (UE) occurred. (DOC) [file pone.0139864.s002.doc]

**Supplement 1. The number of subjects in each group by the days of mechanical ventilation use before the unplanned extubation (UE) occurred.**

| **Days** | **UE group (n=37)** | **Non-UE group (n=156)** |
| --- | --- | --- |
| 1 | 5 | 20 |
| 2 | 6 | 26 |
| 3 | 4 | 17 |
| 4 | 5 | 21 |
| 5 | 2 | 9 |
| 6 | 1 | 5 |
| 7 | 3 | 13 |
| 8 | 6 | 25 |
| 11 | 2 | 8 |
| 12 | 1 | 4 |
| 13 | 1 | 4 |
| 19 | 1 | 4 |

We randomly selected the non-UE group from 1,738 non-UE patients based on one UE subject for four non-UE subjects policy. The data of the non-UE group at the corresponding day of UE incident were then collected.
