# Supplementary material for: Revisiting Unplanned Endotracheal Extubation and Disease Severity in Intensive Care Units
Source: PLoS One. 2015 Oct 20;10(10):e0139864. doi: 10.1371/journal.pone.0139864 (PMC4617893; doi:10.1371/journal.pone.0139864)
Supplement: S2 File — (DOCX) [file pone.0139864.s003.docx]

**Supplement 2. A. Hazard Ratio for mortality using Cox regression:**

| Hazard Ratio | n=190 | Subset |
| --- | --- | --- |
| UE | 0.821 | 0.757 |
| APACHE_II | 1.06* | 1.043* |
| age | 1.014 | 1.033* |
| hepatobiliary_pancre_0_1 | 2.303* | 1.856^#^ |
| shock_0_1 | 2.612* | 2.536* |
| bact_specific_0_1 | 0.448* | 0.667 |
| skin_infection__soft_0_1 | 0.3^#^ | 0.326^#^ |
| vascular_0_1 | 1.409 | 1.629 |
| F_I_O_2____ |  | 15.208* |

Note: Pancre: pancreatic disorders, _0_1: 0 for no, 1 for yes, soft: soft tissue, vascular: vascular disorders, F_I_O_2_: fraction of inspired O_2_. *p <0.05, ^#^ indicating 0.05<p<0.1, and the others are insignificant.

**Supplement 2. B. APACHE II and the related factors: Differences of APACHE II scores between having and having no shock.**

| Shock | APACH II |
| --- | --- |
| no | 24.2±9.1 |
| yes | 27.5±8.3 |
| p value* | 0.014 |

*t-test

**Relationship between age or F_I_O_2_ and APACHE II scores**. Upper panel: r=0.24, p =.002; Lower panel: r= 0.23, p= .001
