# Supplementary material for: Revisiting Unplanned Endotracheal Extubation and Disease Severity in Intensive Care Units
Source: PLoS One. 2015 Oct 20;10(10):e0139864. doi: 10.1371/journal.pone.0139864 (PMC4617893; doi:10.1371/journal.pone.0139864)
Supplement: S3 File — The scores (mean±SD) of various disease entities. (DOC) [file pone.0139864.s004.doc]

**Supplement 3. Acute physiological and chronic health evaluation II scores (mean±SD) of various disease entities.**

| **Disease entity or management** |  | **N = 193** | |  | **Score** | | P value |
| --- | --- | --- | --- | --- | --- | --- | --- |
|  |  | Yes | No |  | Yes | No |  |
| Respiratory infection |  | 143 | 50 |  | 26.4±8.6 | 22.7±9.3 | 0.01 |
| Physical restraint |  | 68 | 125 |  | 22.8±7.7 | 26.9±9.3 | <0.01 |
| Pleural disorders |  | 24 | 169 |  | 23.3±6.3 | 25.8±9.3 | 0.1 |
| Coronary artery disease |  | 18 | 175 |  | 23.4±8.1 | 25.7±9 | 0.3 |
| Genitourinary disorders |  | 27 | 166 |  | 23.6±9.1 | 25.8±8.9 | 0.2 |
