# Supplementary material for: Revisiting Unplanned Endotracheal Extubation and Disease Severity in Intensive Care Units
Source: PLoS One. 2015 Oct 20;10(10):e0139864. doi: 10.1371/journal.pone.0139864 (PMC4617893; doi:10.1371/journal.pone.0139864)
Supplement: S4 File — The process of weaning from the MVS used in this institution. (DOC) [file pone.0139864.s005.doc]

**Supplement 4. The weaning protocol**

The process of weaning from the MVS was started when the initial indications for intubation were reversed, if the hemodynamics of the patient were stable with noradrenaline <1 μg/min, or if oxygenation was adequate with the MVS using a fraction of inspired oxygen (F_i_O_2_) of ≤0.4. Acceptable weaning indexes included rapid shallow breathing index ≤105, tidal volume >6 mL/kg, breathing frequency <30 b/min, minute ventilation <10 L/min, peak inspiratory and expiratory pressures >+20 and <-20 cm H_2_O, respectively, and cuff-leak volume >105 mL for planned extubation, and these indexes were routinely measured every 1-3 days. When the pressure support level of MVS fell to ≤8-10 cm H_2_O and the patients’ cardiopulmonary responses were adequate (heart rate 60-120 beat/min, systolic blood pressure 90-200 mm Hg, breathing frequency 10-30 breath/min, minute volume 5-10 L/min, and stable breathing pattern), extubation was performed after the ICU physician and respiratory therapist reached a consensus. However, the final decision was made by the ICU physician or the combined in-charge surgeons.
